# Ultrastructure for the diagnosis of primary ciliary dyskinesia in South Africa, a resource-limited setting

**DOI:** 10.3389/fped.2023.1247638

**Published:** 2023-08-14

**Authors:** Monica Birkhead, Samuel Otido, Theodore Mabaso, Keketso Mopeli, Dorcas Tlhapi, Charl Verwey, Ziyaad Dangor

**Affiliations:** ^1^ Centre for Emerging Zoonotic and Parasitic Diseases, National Institute for Communicable Diseases – a Division of the National Health Laboratory Service, Johannesburg, South Africa; ^2^ Department of Paediatrics and Child Health, Aga Khan University Hospital, Nairobi, Kenya; ^3^ Department of Paediatrics and Child Health, Faculty of Health Sciences, Chris Hani Baragwanath Academic Hospital, University of the Witwatersrand, Johannesburg, South Africa; ^4^ Medical Research Council: Vaccines and Infectious Diseases Analytics Research Unit, University of the Witwatersrand, Johannesburg, South Africa

**Keywords:** primary ciliary dyskinesia, clinical phenotype, ultrastructure (electron microscopy), resource-limited, South Africa

## Abstract

**Introduction:**

International guidelines recommend a multi-faceted approach for successful diagnoses of primary ciliary dyskinesia (PCD). In the absence of a gold standard test, a combination of genetic testing/microscopic analysis of structure and function/nasal nitric oxide measurement is used. In resource-limited settings, often none of the above tests are available, and in South Africa, only transmission electron microscopy (TEM) is available in central anatomical pathology departments. The aim of this study was to describe the clinical and ultrastructural findings of suspected PCD cases managed by pediatric pulmonologists at a tertiary-level state funded hospital in Johannesburg.

**Methods:**

Nasal brushings were taken from 14 children with chronic respiratory symptoms in keeping with a PCD phenotype. Ultrastructural analysis in accordance with the international consensus guidelines for TEM-PCD diagnostic reporting was undertaken.

**Results:**

TEM observations confirmed 43% (6) of the clinically-suspected cases (hallmark ultrastructural defects in the dynein arms of the outer doublets), whilst 57% (8) required another PCD testing modality to support ultrastructural observations. Of these, 25% (2) had neither ultrastructural defects nor did they present with bronchiectasis. Of the remaining cases, 83% (5) had very few ciliated cells (all of which were sparsely ciliated), together with goblet cell hyperplasia. There was the apparent absence of ciliary rootlets in 17% (1) case.

**Discussion:**

In resource-limited settings in which TEM is the only available testing modality, confirmatory and probable diagnoses of PCD can be made to facilitate early initiation of treatment of children with chronic respiratory symptoms.

## 1. Introduction

Early diagnosis of primary ciliary dyskinesia (PCD) is of paramount importance for affected children's morbidity and longevity (1), particularly given the subsequent treatment and management demands on resource-limited, healthcare settings. The heterogeneous clinical symptoms that combine to give a clinical and diagnostic PCD phenotype, require a multifaceted laboratory approach, given that a single testing modality is unlikely to provide a definitive answer for all cases (2–4). All the relevant diagnostic testing [nasal nitric oxide measurements, high speed video microscopy, genetic testing, transmission electron microscopy (TEM), epithelial air:liquid interface culturing, and immuno-fluorescence microscopy] requires specialised equipment and expertise (4, 5), a fact that impacts negatively on the use of comprehensive laboratory testing for PCD diagnoses in countries with overburdened/resource-limited healthcare facilities (6, 7). Despite being an upper-middle income country, South Africa has a high Gini coefficient of 0.63, and more than 80% of the population depend on state-funded healthcare facilities (8, 9). Rumman et al. (10, 11) evaluated options for PCD diagnoses in resource-limited countries, reporting that any reduction in the technical standard of the equipment/method used resulted in a lack of sensitivity, and that there was still a fundamental reliance on expertise, specialised equipment, and collaboration with suitably equipped groups having this expertise. As would be expected then, nationwide specialised centres for the diagnoses of relatively rare, non-communicable diseases, are not evident in the South African setting. This situation is unlikely to change in the near future, given the high burden of tuberculosis and retroviral disease, and the continuing socio-economic reverberations of the COVID pandemic (12).

Standardised reporting of ultrastructural findings was only possible due to the development and publication of international consensus guidelines for reporting TEM-PCD results (13). The presence of Class 1 ciliary defects (involving outer dynein arms (ODA), outer and inner dynein arms (O + IDA), and microtubular disarrangement with inner dynein arm loss) in more than 50% of transverse sections through a minimum of 50 epithelial cilia, are considered confirmatory in clinically-suspected cases of PCD. Secondary ciliary defects can only be categorized as Class 2 PCD ultrastructural defects after a confirmatory result has been obtained using another PCD-testing modality, because although ultrastructural changes such as ODA + IDA defects in 25%–50% of cilia, central complex defects, and the presence of few or no cilia with mis-located basal bodies, can be suggestive of a case of PCD, these defects can also result both from acute or chronic infections, and from epithelial inflammation caused by asthma, pollutants, or smoking. As secondary ciliary defects may be transient and reversible once the antagonist has been removed, a repeat brushing for TEM may confirm the necessity for alternative PCD testing in some cases (5, 14). The limitations of all PCD diagnostic tests have been reviewed most recently by O'Connor et al. (3).

TEM as a PCD diagnostic, is considered unsuitable in resource-limited settings because it depends upon proficient specimen collection, requires expensive infrastructure and analytical expertise, and is technically laborious (10). Additionally, the diagnostic sensitivity of TEM alone can never exceed 70%, given that ciliary ultrastructure appears normal in up to 30% of PCD patients with two proven bi-allelic or X-linked hemizygous mutations in PCD-related genes (2). However, TEM is the only routinely available PCD laboratory tool in the tertiary academic hospitals of the South African public healthcare system. Nasal brushings were taken from suspected PCD patients presenting to a pediatric pulmonology ward of a large public hospital, and submitted for TEM analyses in order to compare the clinical and ultrastructural findings of these cases.

## 2. Materials and methods

### 2.1. Study population and design

**Figure 1:**
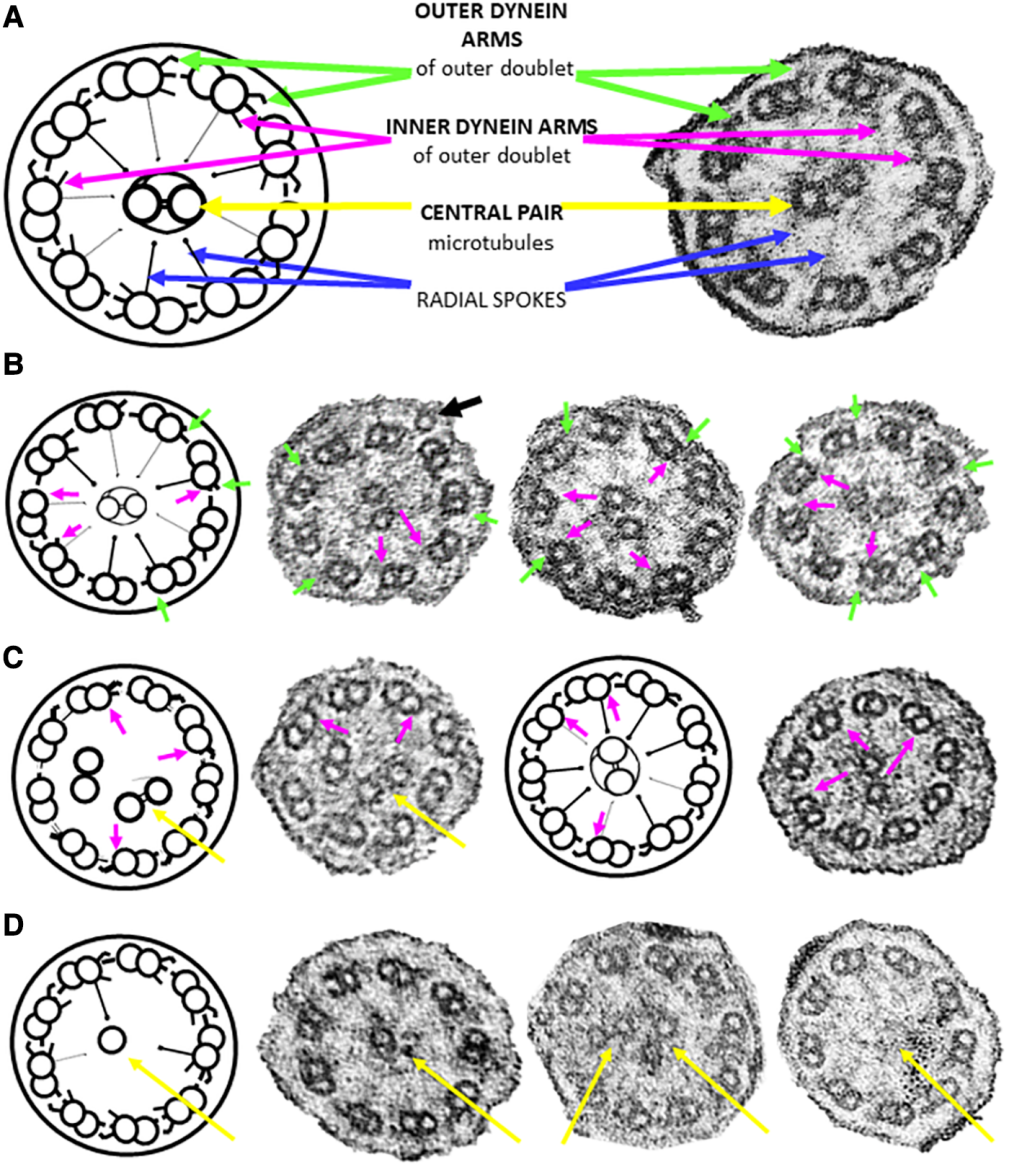
Ciliary cross sections (schematic and micrographs) through the median portion of ciliary axonemes from nasal brushings. (**A**) Normal ultrastructure highlighting noteworthy features used in this study. Each of the nine outer doublets has an outer (green arrow) and an inner (magenta arrow) dynein arm. The doublets are connected to the central pair of microtubules (yellow arrows) by radial spokes (blue arrows). (**B**) Class 1 defects involving the truncation/absence of outer (green arrows)/inner (magenta arrows) dynein arms. Note a peripheral supernumerary microtubule in the first photomicrograph (black arrow). Not all defects are arrowed. (**C**) Class 1 defect of microtubular disorganisation (8 peripheral doublets with the ninth doublet in the central area of the axoneme together with the central pair) and missing inner dynein arms (magenta arrows). (**D**) Secondary ciliary defects involving the central pair of microtubules (either one missing, or an extra one, or none at all distally and in median parts of the axoneme). All cilia are 0.2–0.3 µm in diameter.

Fourteen children less than 16 years of age that underwent nasal brushing for suspected ciliary dyskinesia at the pediatric pulmonology unit of a tertiary-level hospital south of Johannesburg, from 1 May 2019 to the 30 June 2022, were included in this retrospective study. The hospital has approximately 400 pediatric admission beds and various subspecialty services. The pediatric pulmonary subspecialty unit was established in January 2011 and manages patients with complex respiratory problems including PCD. Children may be referred from the general pediatric wards or referral hospitals from southern parts of Gauteng and the North West province. For this retrospective study, nasal brushings of three, unrelated, healthy people were also examined as controls (Figure 1A), to provide some indication of defect frequency (15), albeit non-representative of any particular South African population group given the small sample size. We retrospectively reviewed clinical records, which were paper-based, maintained in the pediatric pulmonology patients' files, and included any electronically-communicated laboratory results, together with the observations made with TEM. All data were anonymized.

### 2.2. Standard of care practices

Children with chronic oto-sino-pulmonary disease, chronic suppurative lung disease, bronchiectasis, persistent wet cough or recurrent chest infections, or situs inversus, are referred to the pediatric pulmonologists for further investigations, including for PCD. Investigations include a computerized tomography (CT) scan, bronchoscopy with lavage, and blood and lung function tests. Pulmonologists collected various clinical data including gender, age of patient, date of first visit, the development of bronchiectasis, and where possible, a PICADAR score (**P**r**i**mary **C**ili**ar**y **D**yskinesi**a R**ule). This scoring system was developed to rate the likelihood of PCD in children with chronic wet coughs (16), with scores based on the presence or absence of the following: term pregnancy, neonatal respiratory symptoms, admission to a neonatal unit, situs inversus, congenital cardiac defects, persistent perennial rhinitis, and chronic ear or hearing problems. Scoring of clinical features using the American Thoracic Society (ATS) PCD guidelines (17) was also done. Nasal brushings were taken from the inner turbinates using a flexible, nylon laparoscopy brush with a twisted wire shaft (WS-1812XA3, Wilson instruments, Shanghai, China). If these brushes were not available, a small cervix brush with the fibres trimmed off, was used. Of fourteen patients, eight required a single brushing, while only two of the six requiring second brushings received them.

### 2.3. Transmission electron microscopy

Brushings were immediately placed in buffered glutaraldehyde (2.5% EM grade glutaraldehyde, in 0.1 M sodium cacodylate buffer [Sigma-Aldrich purum ≥98%, CAS# 6131-99-3, pH 7.01, osmotically adjusted by the addition of 0.09 M sucrose (Sigma, CAS# 57-50-1, for molecular biology), 0.01 M magnesium chloride (Merck EMSURE® CAS# 7791-18-6), and 0.01 M calcium chloride (Merck EMSURE® CAS# 10035-04-8)] and stored in a fridge for EM processing. For processing, the brushes were immersed in fresh fixative in a petri dish, and gently cleaned of all adherent matter under a dissecting microscope. Collected fragments were rinsed in buffer (3 × 30 min) with gentle centrifugation for pelleting when necessary (10 min at 500× g), then post-fixed in 1% buffered osmium tetroxide (SPI-Chem CAS#2 0816-12-0) for one hour, buffer rinsed (3 × 30 min) with a final rinse in pure water (5 min) prior to dehydration in a graded ethanol series (10%, 30%, 50%, 70%, 90%, 100%) at 30 min intervals. Three rinses of absolute ethanol were performed before resin infiltration (resin: ethanol 1:3, 1:1, 3:1, pure resin) using Agar scientific low viscosity resin® following manufacturer's instructions. After a third pure resin change which was left overnight, the specimens were transferred to gelatin or BEEM® capsules, polymerised at 70°C, sectioned at 70 nm on a Leica EM-UC6 ultramicrotome, and double stained with aqueous 4% uranyl acetate (15 min) followed by Reynold's lead citrate (10 min). Grids were viewed at 120KV on an FEI Tecnai Spirit TEM (ThermoFisher, Oregon, USA, formerly FEI, Eindhoven, the Netherlands) and imaged using an Olympus Quemesa CCD camera (EMSIS GmbH, Germany). Electron microscopy was performed at the National Institute for Communicable Diseases (RRID:SCR_022662 NICD). No tomography/cryotomography facilities were available.

The consensus guidelines for TEM-PCD diagnostic reporting (13) were followed. TEM observations of nasal brushings done prior to these guidelines, were reviewed and updated for compliance where possible. Ultrastructural defects in ≤10% of cilia were considered to be within the normal range (14). Other ultrastructural features mentioned in individual reports in the literature as suggestive of PCD, such as axonemal asymmetry in confirmed PCD cases showing no other ciliary defects (18), and ciliary inclusion disease (19), were also noted. General features of respiratory epithelium that are not necessarily indicative of PCD, such as cell bulging which results in a non-planar epithelial surface (20, 21) and ratios of ciliated cells to goblet cells (22) were also recorded. To date, the development of the necessary expertise in ultrastructural analysis has been supported by Dr Amelia Shoemark of the University of Dundee.

### 2.4. Statistical analysis

Clinical data and ultrastructural observations were compared for suspected PCD cases. Categorical variables were reported as proportions. Continuous variables which were not normally distributed, were presented as medians and inter-quartile ranges.

## 3. Results

### 3.1. Clinical data

Of the 14 children that were selected for nasal brushings, twelve (86%) presented on the first clinical visit with recurrent respiratory tract infections, one infant suffered respiratory distress in the neonatal period, and one child presented with pulmonary tuberculosis (Supplementary Table S1). The age at first visit ranged from four weeks to ten years, with a median age of 2.5 years (inter-quartile range 18.5 months – 5 years 4.5 months), and eight (57%) were male. Situs abnormalities were recorded in four (29%) children and a further one reported dextroposition. Bronchiectasis was present in eight (58%) children, not confirmed in two (4%) children, and absent in the three (21%) children who were under eleven months on their first clinic visit. Available PICADAR scores varied from 2 to 13, with six children (43%) having a score ≥7. ATS scores mirrored the PICADAR scores in all but one patient (possible PCD by ATS, unlikely on PICADAR).

### 3.2. Transmission electron microscopy

Sufficient epithelium for ultrastructural analyses was found in all brushings. Six (43%) of the 14 specimens exhibited Class 1 defects (Table 1, Supplementary Table S1 and Figures 1B,C), of which five involved both outer and inner dynein arm truncation or absence, whilst only one case of Class 1 inner dynein arms and microtubular disorganization was recorded. Ciliary profiles of a minimum of 11 different cells were examined in these Class 1 cases. Two (14%) of the 14 patients had normal ciliary ultrastructure, so axonemal asymmetry assessments were made, as Blanco-Máñez et al. (16) identified 35% of PCD cases with normal ultrastructure using this method. Both cases demonstrated that more than 40% of the cilia had symmetry breaks in the β-tubules of the outer doublets. Only one (7%) of the 14 study cases had rare cells with ciliary inclusion disease (and Class 1 defects).

**Table 1:** Detailed ultrastructural observations of nasal brushings from suspected PCD cases.

Case	Class 1 defects	Secondary ciliary defects	Other non-defining ciliary defects and observations
1	ODA (+IDA)	–	Abundant supernumerary microtubules, with a maximum of 12 peripherally and 3 centrally; <0.2% multiple axonemes; rare ciliary inclusion disease (3 seen); epithelial cells mostly planar
2	IDA and microtubular disorganisation	–	9 + 2 not radially symmetrical in <0.5% of profiles; 6% with one central microtubule; 3% with 8 + 1 doublets; 24% with 8 + 1 doublets and a central pair; extra microtubules: centrally 17%, peripherally <1%; several compound axonemes. Compacted mucus with embedded necrotic cells, cellular debris and bacteria; The surface of the ciliated cells not planar, generally conical.
3	ODA + IDA	–	Extra microtubules: centrally 2%, peripherally 1%; central pair absent 3%.
4	ODA (+IDA)	–	Many conical cells; possibly radial spoke malformation; 2 lacking central doublets, 1 single peripheral, 1 multiple microtubules peripherally, some distorted outer ciliary membranes;
5	ODA + IDA	–	Extra microtubules: 6% centrally, 2% peripherally; 1% 8 + 1; No normal ciliary profiles seen at all. Other epithelial features normal.
6	ODA + IDA	–	Extra microtubules: centrally 1%, peripherally 3%; compound axonemes 4%. Epithelial cells generally planar, evenly ciliated, normal ciliated:goblet ratio; many conical cells; signs of infection (neutrophils, replicating bacteria);
7	–	–	No ciliary defects along entire length of cilia. Extra microtubules: centrally 2, peripherally 3; central pair absent 1. Radial spokes normal.
8	–	–	No ciliary defects along entire length of cilia. <4% with ODA + IDA absence; extra microtubules centrally ∼1%.
9	–	Few ciliated cells ? RGMC	>8 goblet cells for every ciliated cell; very few ciliated cells all sparsely ciliated; basal bodies scattered in cytoplasm/irregularly docked on membrane
10	–	Insufficient No ciliated cells	Maximum of 19 goblet cells:0 columnar cells (non-ciliated) over 114 µm
11	–	Insufficient No ciliated cells	Maximum of 10 goblet cells:0 columnar epithelial cells (no cilia seen)
12	–	Few ciliated cells ? RGMC	Ratio of goblet:ciliated cells in epithelial strips from 5:0 to 8:1 to 18:0. Misplaced basal bodies in one cell section, complete absence of cilia in another, and when present, there was a reduced number of cilia, with only one cell having >20 associated ciliary profiles. Of only 12 suitable ciliary profiles seen in cross section: extra microtubules: centrally 3, peripherally 2; multiple axonemes 4.
13	–	Few ciliated cells ? RGMC	Sparse ciliation (7 cilia/sectioned cell), with a ratio of goblet:ciliated cells of 4:1. Basal bodies not always docking at the cell membrane; No striated connective fibres (rootlets) attached to the basal bodies or evident in the cytoplasm. Of the 17 suitable ciliary cross sections seen, 8 had ODA defects, 2 ODA + IDA defects, 1 had no central pair, another had 8 + 1 doublets, 3 had extra microtubules (centrally 2, peripherally 1).
14	–	>20% of ciliary profiles lacking one central microtubule	Needs second brushing and other confirmatory testing
C1 C2 C3	–	–	Maximum defects in control brushings: ODA/+IDA 6%; central complex 8%; supernumerary microtubules 4%; <1% compound axonemes; conical cells max. 32%. Lowest goblet:ciliated ratio 1:4

? RGMC = possible case of reduced generation of multiple ciliated cells: basal body orientation, reduced number of ciliated cells, and cilia per cell, but requires genetic testing.

**Figure 2:**
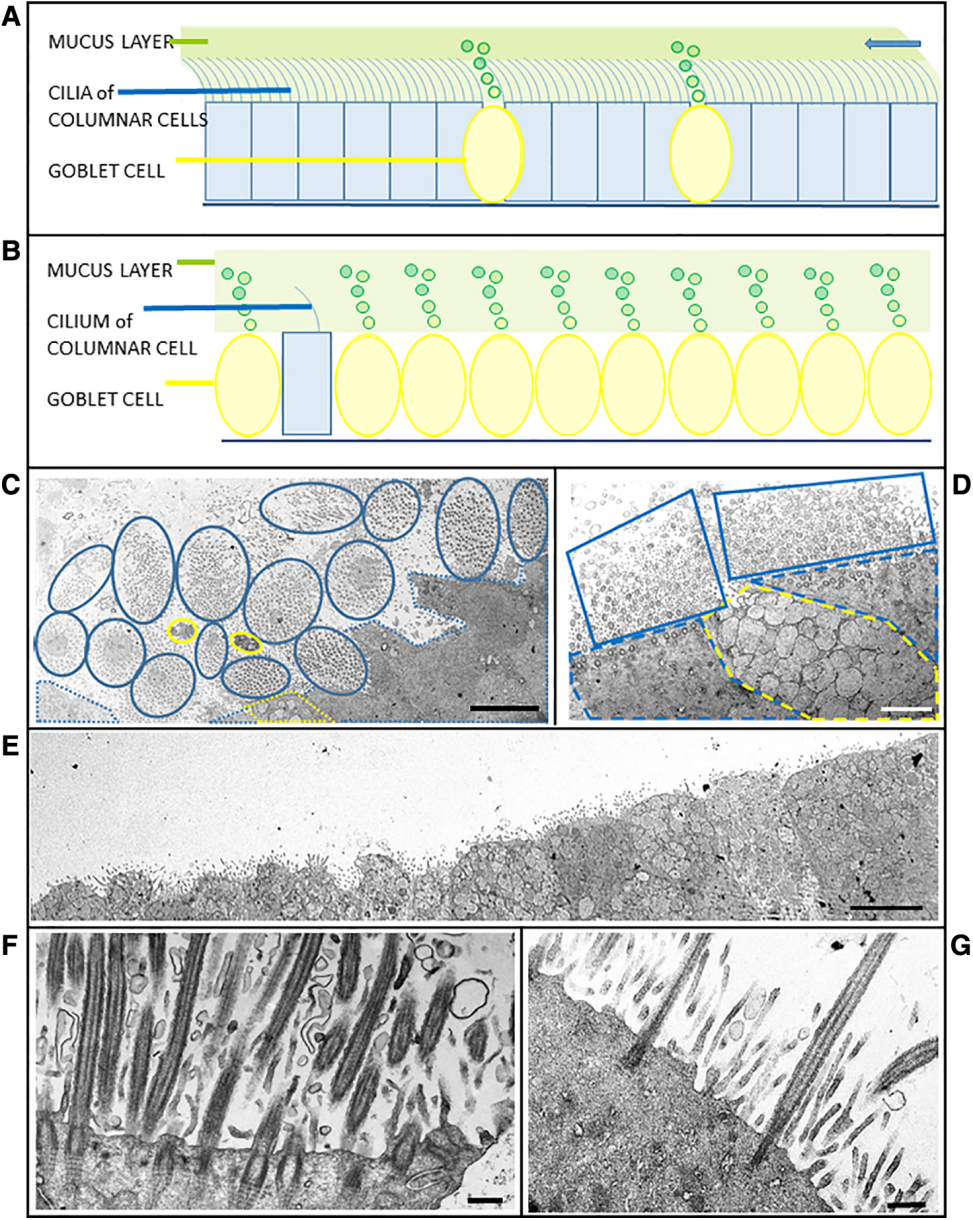
Schematics and case micrographs of sections through epithelial cells from nasal brushings. (**A**) Normal pseudostratified epithelial layer with a 5:1 ratio of multi-ciliated columnar cells to mucin-producing goblet cells. The cilia beat synchronously to move the overlying mucus (and any embedded allergens/pathogens) out of the airways (arrow). (**B**) Epithelial layer illustrating goblet cell hyperplasia, with rare ciliated columnar cells having reduced numbers of cilia. Lack of ciliation results in accumulation and stasis of the overlying mucus layer. (**C**) Tangential transverse section through the apices of conical, ciliated columnar cells. Each blue encircled clump of cilia represents one columnar cell. Goblet cells are demarcated in yellow. Although epithelial layers should be planar, the ratio of ciliated:goblet cells is normal. (**D**) Another tangential section through a goblet cell (yellow dotted line) between two ciliated cells (blue dotted lines). The associated cilia (solid blue lines), which are mostly in cross section, are abundant and evenly spaced. (**E**) Micrograph illustrating goblet cell hyperplasia (possible case of reduced generation of multiple motile cells) in an epithelial strip in which no ciliated cells are evident. (**F**) Section through a typical ciliated columnar cell, with numerous ciliary profiles interspersed with microvilli. Note the striated rootlets extending from the basal bodies into the cytoplasm. (**G**) Section through a columnar epithelial cell illustrating oligocilia - sparsely ciliated with a distinct layer of microvilli. Scale bars: C = 5 µm; D = 2 µm; E = 5 µm; F = 0.5 µm; G = 0.4 µm.

Secondary ciliary defects were observed in six (43%) patients. Of these, there was only one case of anomalies in the central pair of microtubules (Figure 1D), with the five remaining cases being characterised by very few, sparsely-ciliated columnar epithelial cells, as well as goblet cell hyperplasia (a disproportionate ratio of goblet cells to ciliated columnar cells). Observations included intact epithelial strips of 38–52 µm with four to eight goblet cells per ciliated cell, and in one case, no ciliated cells at all in an epithelial strip of 105 µm, with 19 contiguous goblet cells (Figures 2A–G). Of these five cases of oligocilia, two were consistent on repeat brushings several months apart, but second brushings were not available for the remaining three, in one case because the patient refused. Basal bodies displaced from the surface of the cell were also apparent in the cytoplasm of two of these cases (suggestive of the Class 2 defect of reduced generation of multiple ciliated cells – RGMC), and in only one case, basal body rootlets (Figure 2F) were not visible in any examined sections.

Misshapen ciliated cells which resulted in a non-planar epithelial layer, were more frequent in suspected cases (>40% of cells appeared conical) than in any of the three controls (two of which had fewer than 15% conical ciliated cells, with the third control brushing having <32%). All three controls exhibited less than 9% ciliary defects, and had a ratio of 1:4 goblet:ciliated cells.

## 4. Discussion

In resource-limited settings, patient morbidity and mortality are frequently dependent on experienced clinicians alone. For diseases like PCD, which present clinically with a high degree of phenotypic variation, the burden of responsibility on the clinicians is further exacerbated. This study sought to evaluate TEM in the early diagnosis of PCD, given that early diagnosis can direct both the initiation of treatment (in order to reduce recurrent infections, lung damage, the rate of disease progression, and the development of bronchiectasis) and of appropriate cost-effective management of PCD patients. Although the lack of any other PCD diagnostic test limited the direct comparisons that could be made between individual clinical diagnoses and the corresponding TEM observations, clinical suspicion of PCD and ciliary ultrastructure overall appeared concordant in 43% of the cases. This value compares well with other recent PCD-TEM-only reports from resource-limited settings, for example 17.9% in an Iranian study (23), 34% in the Palestinian cohort (11) and 43% (all cases) −70% (only cases with high clinical suspicion) in a Brazilian study (6). These studies were based on large numbers of clinically suspected PCD cases of all age groups, in contrast to the current small pediatric study in which correlation between clinical and ultrastructural data was probably due to the pediatric pulmonologists' heightened awareness of PCD as part of a diagnostic differential, to the small sample size, and to the lack of any other test to corroborate the clinical - TEM findings.

The majority of patients in this study presented after one year of age, with all patients older than a year on their initial visit, with completed TEM observations that confirmed or supported a possible PCD diagnosis, having bronchiectasis. Two patients over the age of five years, that did not have bronchiectasis on initial visits, also lacked any apparent ciliary ultrastructural defects. The axonemal asymmetry assessments of these two patients' brushings appeared to support the clinical phenotype, with the percentage of cilia with β-tubule displacement exceeding 40% in both cases. However, given the limitations of this diagnostic approach (which include highly subjective interpretation and overlap between the extent of axonemal asymmetry in control and PCD-confirmed cases), and the fact that asymmetry was less apparent in the median and proximal portions of cilia, this assessment needs to be ratified by international consensus. As 30% of PCD patients do not have ciliary defects visible on conventional TEM, these two possible cases require alternative testing: most appropriately for a resource limited setting, would be the use of light microscopy for basic observation of ciliary movement, although international guidelines suggest either genetic testing, nasal nitric oxide measurement, or high speed video microscopy of epithelial strips/immunofluorescence microscopy (3, 10). Genetic testing is also not conclusive in a third of the ∼30% of PCD cases with normal ultrastructure (3, 24), but testing should be at least of the genes known to be associated with normal ultrastructure in PCD, preferably whole exome sequencing as well (2, 3). Both patients presented clinically with typical PCD phenotypes: recurrent infections; high PICADAR and ATS scores; and situs abnormalities. Situs abnormalities do manifest in non-PCD patients too (25), but in this study, of the three additional patients that exhibited abnormal laterality, two had Class 1 ultrastructural defects, and one had secondary ciliary defects warranting a second brushing and an alternative test. Four of the six patients who had TEM corroboration of clinically-suspected PCD, did not present with laterality defects, which numbers support current literature reports of approximately half of all PCD cases having situs abnormalities (3, 5).

The mislocation of basal bodies in specimens with few ciliated cells and a reduced number of cilia per cell, is considered a secondary ciliary defect, as normal ciliary features may recur. This restoration may take longer than previously thought, given the observation of RGMC in epithelial cells of SARS-CoV-2 patients up to a year post-infection (26). The periodicity of repeat brushings needs to be taken with this consideration in mind, although in the current study setting, second brushings were frequently unavailable.

Recurrent respiratory tract infections contribute towards a PCD phenotype (27). Goblet cell hyperplasia (with the associated mucus hyper-production/hyper-concentration, impaired mucociliary clearance and increased susceptibility to infection), is frequently described in chronic pulmonary disorders (non-cystic fibrosis bronchiectasis, idiopathic pulmonary fibrosis, cystic fibrosis, asthma, chronic obstructive pulmonary disease) but is rarely mentioned in ultrastructural observations of suspected PCD cases (28, 29) – possibly either because the focus is on the cilia and not the mucins, or the fact that in this study, four of the five cases of secondarily defective cilia with goblet cell hyperplasia had proven bronchiectasis (an indication of iterative infections) before brushings were taken for TEM. A dearth of ciliated cells and an abundance of goblet cells involves activation of the Notch signalling pathway, as this determines the differentiation of secretory cells from basal cells. Conversely, Notch inhibition is necessary for the formation of multiciliated cells, so the Notch signalling pathway is integral to the development of both RGMC and goblet cell hyperplasia (2). Mutations in the genes known to cause the Class 2 defect of RGMC are not associated with laterality defects, and in this study, none of the five cases with oligocilia had situs abnormalities. The scarcity of cilia could have contributed to the apparent absence of striated rootlets in one suspected case, but usually the rootlets (and associated protein, rootletin) of all ciliated/flagellated cells are highly conserved, offering structural support and signal mediation for ciliary functioning (30). Not unexpectedly then, genetically altered mice with lung mutations resulting in the absence of basal body rootlets, suffered insufficient mucociliary clearance (31). Ultrastructural observations in the present study could be supported by genetic testing for the *CROCC* (Ciliary Rootlet Coiled-Coil, Rootletin) gene, or by rootletin immunofluorescence microscopy of epithelial strips. The initial clinical presentation of this patient was for pulmonary tuberculosis, but the possibility of ultrastructural defects being long-term sequelae would be an interesting research avenue to explore, particularly given the importance of this disease in post-infectious development of bronchiectasis in Sub-Saharan Africa (32).

The sixth case with secondary defects (missing microtubule/s in the central complex) did not have proven bronchiectasis, but dextroposition and neonatal chest symptoms with admission to the neonatal ward, so the PICADAR and ATS scores were correspondingly high. Although it is tempting to consider this to be a possible case because of the concordance between clinical and TEM observations, it must be remembered that all secondary ciliary defects can only be suggestive of PCD (not diagnostic), and require genetic testing to confirm a diagnosis. However, this possible case corroborates other reports published before the international TEM consensus guidelines, of the abnormal central complex being a marker of disease severity (33).

In the validation of the PICADAR tool, 18.7% of PCD-confirmed patients had a score ≤5 (16). Low PICADAR scores were recorded for a number of our patients, two with Class 1 ultrastructural defects, and three of the secondarily defective cases. ATS and PICADAR scores generally corresponded, with ATS including one of the Class 1-low PICADAR patients. One of the suspected secondarily defective cases with few ciliated cells had very high clinical scores which contrasted with the other three cases for which sufficient information was available, suggesting that diagnoses of PCD in these three cases might be less likely. Insufficient clinical information was available for scoring of one of the secondarily defective cases, which highlights one of the limitations of this study: retrospective studies rely on stored data sets that may be incomplete for a variety of reasons. For example, in our setting, it is not always the child's guardian who is able to bring the child to the hospital, so detailed information/history is often unavailable, and contact details of some guardians were no longer valid on follow-up. Other difficulties experienced in this study were the time taken to do thorough ultrastructural analyses, and the cost of maintaining the TEM (particularly with national power supply interruptions).

Most large tertiary academic hospitals in South Africa have TEM routinely available, which is thus the sole diagnostic testing equipment for PCD nationally, given the challenges within the health system. Development of human resources with the necessary microscopy expertise would be a pre-requisite to the application of TEM diagnostic testing at these facilities. If available, TEM is a valuable addition to clinical diagnoses, irrespective of the diagnostic limitations inherent in this testing method, and it may be possible to improve on conventional TEM inputs using PCD-specific software applications (34, 35). Despite the small scale of this study, it has contributed the first report on PCD diagnostics in South Africa, and it has aided in the development of the database for the BACPAC Network (Bronchiectasis in African Children: Prevalence, Etiology and Clinical spectrum) established in 2020 (32).

## Data Availability

The original contributions presented in the study are included in the article/**Supplementary Material**, further inquiries can be directed to the corresponding author.
